# Coping of Chronically-Ill Patients during the COVID-19 Pandemic: Comparison between Four Groups

**DOI:** 10.3390/ijerph20064814

**Published:** 2023-03-09

**Authors:** Mateusz Łuc, Marcin Pawłowski, Arkadiusz Jaworski, Karolina Fila-Witecka, Dorota Szcześniak, Hanna Augustyniak-Bartosik, Dorota Zielińska, Aleksandra Stefaniak, Anna Pokryszko-Dragan, Justyna Chojdak-Łukasiewicz, Magdalena Krajewska, Tomasz Pawłowski, Jacek C. Szepietowski, Joanna Rymaszewska

**Affiliations:** 1 Department of Psychiatry, Wroclaw Medical University, 50-367 Wroclaw, Poland; 2 Students Research Association, Department of Psychiatry, Wroclaw Medical University, 50-367 Wroclaw, Poland; 3 Dialysis Unit, Department of Nephrology and Transplantology, Wroclaw Medical University, 50-556 Wroclaw, Poland; 4 Department of Nephrology and Transplantology, Wroclaw Medical University, 50-556 Wroclaw, Poland; 5 Department of Dermatology, Venerology and Allergology, Wroclaw Medical University, 50-367 Wroclaw, Poland; 6 Department of Neurology, Wroclaw Medical University, 50-556 Wroclaw, Poland

**Keywords:** pandemic, COVID-19, SARS-CoV-2, chronic diseases, psychopathology, coping, chronic kidney disease, multiple sclerosis, psoriasis

## Abstract

In many countries, the COVID-19 pandemic led to healthcare reorganization limiting access to diagnostic or therapeutic procedures for chronically-ill patients. In this article, we describe the psychological consequences and coping strategies of several groups of chronically-ill patients. During the cross-sectional survey conducted in 2020, we enrolled 398 patients with four different chronic conditions (psoriasis, multiple sclerosis, and patients who have undergone a kidney transplant or received dialysis). The study sample was examined regarding the experienced stress levels (Perceived Stress Scale) and coping strategies (Brief-COPE). All four groups of patients most commonly declared using problem-focused coping strategies and least commonly reported the use of avoidant coping. Higher levels of perceived stress strongly correlated with self-blaming. The participants who declared previous psychiatric treatment or psychotherapy were more likely to use self-blaming, behavioral disengagement, substance use, and avoidant coping, while previous psychotherapy additionally correlated with emotion-focused coping. Group comparison identifies patients with a chronic neurological disease, such as multiple sclerosis, at higher risk of a less beneficial coping profile than kidney transplant recipients. Further focus on education and early interventions in at-risk individuals is needed, and widely targeted mental health programs are indicated in order to improve the mental health of patients suffering from chronic diseases.

## 1. Introduction

COVID-19 has quickly spread from its place of origin worldwide, leading to a global pandemic emergency. By the end of 2022, the number of confirmed COVID-19 cases has reached over 649 billion worldwide, with reports indicating insufficient testing and detection in numerous regions [1,2]. Initial preventive actions were aimed at the reduction of viral transmission and therefore alleviating the hospitalization burden in order to avoid the collapse of healthcare systems. Prior to the vaccine development, actions oriented toward the reduction of SARS-CoV-2 transmission initially involved long and recurring lockdowns, travel, and social restrictions and resulted in a major reorganization of daily life. These restrictions took a severe toll on the mental health of citizens, the size of which is yet to be accounted for, along with the long-term consequences of both viral infection and social isolation stemming from introduced restrictions [3,4]. 

In many countries, healthcare reorganization resulted in limited access to diagnostic or therapeutic options for novel cases of other diseases [5]. Additionally, various factors contributed to decreased availability of required medical services needed for the maintenance of chronic diseases. Among these factors are (1) personnel shortage stemming from exposure to SARS-CoV-2 and following quarantines and/or infections; (2) reorganization of clinical wards, with medical professionals being transferred to temporary COVID-19 wards and thereby decreased number of professionals remaining at their standard placement and (3) necessity to secure back-up hospital beds in case of a rising incidence during following COVID-19 waves. These factors required additional effort from patients and eventually resulted in a transient reduction of planned appointments, administered procedures, and check-ups [6,7,8,9]. On the other hand, anxiety related to medical institution visits and potential SARS-CoV-2 infection disabled another group of patients from obtaining regular professional medical care.

Numerous reports illustrate the effects of the pandemic on the mental health of the general population, indicating a large distribution of COVID-19-related anxiety symptoms, with some researchers proposing a separate term, COVID stress syndrome [10]. After the early pandemic phases, the acute stress factor gained chronic character leading to a novel manifestation of pandemic-associated mental disorders. Currently, many researchers report on factors contributing or correlated with mental health outcomes in the general population, in students, or in medical professionals [11,12,13,14]. However, much less is known about the consequences of COVID-19-related psychological strain on vulnerable populations, such as individuals at risk for mental disorders, the elderly, and those frequently requiring services of the health care system—patients treated for chronic diseases, such as neurological disorders, dermatological conditions, or patients undergoing dialyses. The early reports indicate a higher incidence of mental disorders such as depression and anxiety in chronically-ill patients [15]. Additionally, studies that focus mostly on the quality of life of people diagnosed with certain chronic medical conditions suggest that the impact of primary diagnosis significantly varies and depends on the characteristics of the disease [16,17,18]. Our previous research showed that 48% of chronically-ill patients presented clinically significant psychopathological symptoms in the early stages of the pandemic [19]. This scale of mental health burden significantly exceeds the levels in the general population ranging from 14% to 27% during the first wave of the COVID-19 pandemic [20,21], and highlights the importance of the predisposing individual traits and potential differences between groups of patients which have not been described so far. 

Individual strategies for dealing with stress are described as coping and are believed to significantly mediate the varied outcomes of global stress factors, such as the COVID-19 pandemic, on one’s mental health. The impact of this mediation remains difficult to quantify. However, some studies present results indicating that used coping strategies in response to either acute or chronic stressful events may actually be responsible for over 50% of mental health outcomes, such as anxiety, depression, or somatization [22,23]. For example, people dealing with stress via avoidance were found to be more likely to manifest anxiety, depression, or symptoms of an eating disorder, while people who respond to stress with problem-solving are at lower risk of these outcomes [24]. Patients diagnosed with multiple sclerosis and using positive reframing, emotional support, instrumental support, religion, planning, and self-distraction were found to be more likely to exhibit post-traumatic growth [25]. There are several classifications of coping strategies that are based on the action direction towards/from a stressful stimulus or emotion use. What is important, the adaptive and detrimental characteristics of each coping strategy may vary depending on the chronicity of the stimulus, its range, and characteristics but also on external factors, such as societal or cultural differences [26]. 

In this article, we describe the psychological consequences and coping strategies of several groups of chronically-ill patients. We aim to distinguish stress-predisposing characteristics related to ongoing chronic disease or sociodemographic factors and also to indicate beneficial and deleterious coping strategies common in this population. With the rapid occurrence of novel SARS-CoV-2 variants, this knowledge may allow better identification of chronically-ill patients at risk of deterioration of their mental health and implementation of early interventions. Additionally, it may serve for better organization of necessary health care in a potential need of temporary lockdown reintroduction.

## 2. Materials and Methods

### 2.1. Study Design and Settings

We have recruited chronically-ill patients with diagnoses of psoriasis, multiple sclerosis (MS), patients who had had a kidney transplant, and patients with chronic kidney disease receiving dialyses to participate in a cross-sectional survey carried out between May and October 2020. The local Bioethical Committee at the Wroclaw Medical University approved the study (KB-468/2020; KB-469/2020; KB-470/2020; KB-417/2020). For detailed methods and procedures, see Pawłowski et al., 2022 [19].

Participants took part in a survey voluntarily and without financial reward. Participation was anonymous, and data was secured at all stages of the study. Questionnaires were delivered both via an online form and printed format, as a consequence of COVID-19 restrictions and for patients’ safety. We used Computer-Assisted Web Interviewing (CAWI) to conduct an online version of a survey and shared it with patients via websites and profiles of MS Societies and Polish Psoriasis. The printed forms were provided to patients at the University Clinical Hospital in Wroclaw, Poland. In the preliminary section of the survey, respondents filled out an informed consent, so participation and processing of data were possible. The online version required confirmation of informed consent so that respondents only then could continue and submit the questionnaire. In the printed version, the respondents confirmed informed consent by signing the form and completing the survey. The participants were evaluated for perceived levels of stress and employed coping strategies. Demographic variables, as well as pandemic-related data, such as previous quarantines, COVID-19 exposures, or infections, were also contained in the survey. We downloaded the data file from an online survey and transcribed manually the information from paper questionnaires to the database.

### 2.2. Participants

The inclusion criteria for the recruitment were: (1) a previous diagnosis of psoriasis (P), multiple sclerosis (MS), being an adult kidney transplant recipient in the past (T) or undergoing dialysis treatments at the present time (D); (2) age over 18 and (3) providing informed consent to participate. Due to the online version of the survey, no medical documents nor confirmations were required in order to preserve anonymity in P and MS groups. Participants from T and D were recruited only at the hospital. Hence, their medical data and documentation were available in order to confirm diagnoses. Exclusion criteria were: (1) age under 18; (2) inability to provide informed consent, and (3) incomplete survey. Psychological and psychiatric data were collected but did not disqualify from participation.

### 2.3. Measurements & Outcomes Measures

The psychometric tools used in the study were selected by a team of experts from different fields (psychiatry, psychology, neurology, dermatology, and nephrology) and were based on appropriate literature. The survey consisted of the following sections:

Sociodemographic and COVID-19 exposure data.

*The Perceived Stress Scale 10* (PSS-10) allows for the assessment of the experienced stress level. It comprises 10 questions intended to evaluate the subjective level of stress [27]. The questionnaire was validated in the Polish population and deemed satisfactory, with validity scores (Cronbach’s alpha) around 0.8. Respondents marked their answers on a 5-point scale ranging from 0 (never) to 4 (very often). The results from all items were summed up to calculate the final score of the PSS-10. The total score reflects the intensity of perceived stress.

*The Brief Coping Orientation to Problems Experienced Inventory* (Brief-COPE; Polish adaptation: *Juczyński & Ogińska-Bulik, 2009*) allows for the assessment of strategies employed in order to cope with a stressful event [28]. The inventory has 28 items (2 questions per each strategy) and was validated in the Polish population. Participants indicate their answers on a 4-point scale ranging from 0 (almost never) to 3 (almost always), and the sum for each strategy is divided by 2. Fourteen strategies can be grouped into three larger categories: problem-focused (active coping, use of informational support, positive reframing and planning), emotion-focused (emotional support, venting, humor, acceptance, religion, and self-blame), and avoidant (self-distraction, denial, substance use, and behavioral disengagement) coping [29]. 

### 2.4. Statistical Analysis

The statistical analysis of the obtained results was performed with the use of IBM SPSS Statistics v. 26 (SPSS Inc., Chicago, IL, USA) software. All data were assessed for parametric or non-parametric distribution. The minimum, maximum, mean, and standard deviation were calculated, whereas for coping strategies and perceived stress level, parameter distribution was assessed for kurtosis and skewness. Due to the relatively large sample, Kolmogorov–Smirnov test results were calculated. Analyzed variables were evaluated using the Mann–Whitney U test and Spearman correlations. Differences between several groups were assessed by the Kruskal–Wallis 1-way analysis of variance on ranks. We additionally performed the mixed-design analysis of variance with a focus on overall coping strategies in a (3) × 4 model. Simple effect tests with Bonferroni adjustment were calculated. A 2-sided *p*-value ≤ 0.05 was considered to be statistically significant. As incomplete surveys were excluded from the study, no missing data was encountered. 

## 3. Results

### 3.1. Participants’ Characteristics

In the study, 398 participants aged from 18 to 89 (M = 45.74; SD = 17.04) were enrolled. According to their diagnosis, they were divided into 4 subgroups: 95 psoriasis patients, 128 patients with a diagnosis of multiple sclerosis, 102 recipients of kidney transplants, and 73 patients receiving dialyses. There was a moderate predominance of women in the study group (n = 238; 59.8%). More than half of the study participants declared to have a higher education, whereas one-third declared a secondary education. In the early phase of the pandemic, most of the study population did not declare a reduced time of work or change in their responsibilities due to the COVID-19 emergence. Similarly, at that time, most of the participants were not exposed to SARS-CoV-2 at their place of employment, nor had they previously been quarantined. In addition, 18.3% were previously treated by a psychiatrist, and 28% had undergone previous psychotherapy. Measured with the use of PSS-10, 62.4% of participants exhibited a moderate level of perceived stress, while the percentages for high and low intensity were 15.7% and 21.8%, respectively. The detailed characteristics of the study group are summarized in Table 1.

The study groups had dissimilar sociodemographic profiles in terms of sex, age, marital status, psychiatric treatment, psychotherapy, and duration of illness. 

The predominant perceived stress level was moderate in all groups except for psoriasis patients, with more than half of them reporting either low or high intensity of perceived stress. The low-stress level was most commonly observed in psoriasis patients (32.6%) and kidney transplant recipients (28.4%). The high-stress level was more frequently reported by patients with multiple sclerosis (25%).

### 3.2. Coping Strategies in the Studied Population

The studied population reported the most common use of problem-focused coping strategies, such as active coping, planning, and positive reframing. Among emotion-focused strategies, emotional support and acceptance were most likely to be employed by the study participants. On the other hand, the studied group of chronically-ill patients claimed to be less likely to use humor and avoidant strategies, with special emphasis on substance use. The detailed data is provided in Table 2.

### 3.3. The Differences in Coping Strategies between Studied Groups

Due to the varied characteristics of distinct subgroups, we performed the Kruskall–Wallis H test in order to analyze differences in coping strategies between the groups of patients. MS and T groups were significantly more likely to use acceptance compared to the P group. Additionally, they employed active coping more frequently than D patients. The MS group had a higher tendency to self-blame than T and also to use positive reframing and emotion-focused coping more than P. Moreover, MS claimed to use venting and self-distraction more frequently than P and T. 

D employed emotional support more often than P and employed denial more often than P and T. Both MS and D were more prone to behavioral disengagement and avoidant coping than P and T. On the other hand, P and MS reported higher substance use and less religious coping than T and D. The detailed data is provided in Table 3.

We additionally confirmed our results with the mixed-design analysis of variance with a focus on overall coping strategies, problem-focused coping, emotion-focused coping, and avoidant coping in the studied groups. All four groups of patients most commonly declared using problem-focused coping strategies and least commonly reported the use of avoidant coping. 

**Figure 1:**
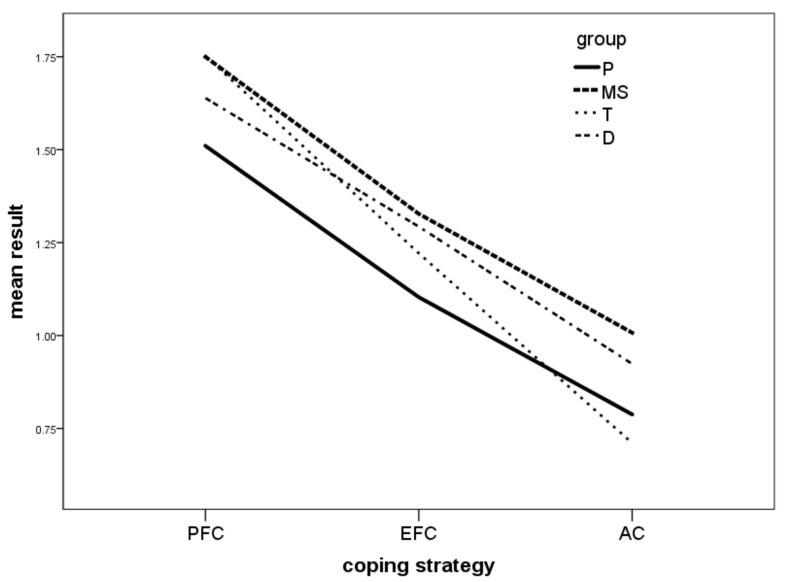
Coping strategies in four groups of patients—interaction effect. P—psoriasis, MS—multiple sclerosis, T—kidney transplant recipients, D—dialysis patients. PFC—problem-focused coping, EFC—emotion-focused coping, AC—avoidant coping.

The P group used problem-focused coping significantly more frequently than MS and T. The MS and D were more likely to employ emotion-focused coping strategies than P. Moreover, MS used avoidant coping more commonly than P and T, whereas T were also less likely to use avoidant coping than D (Figure 1). Additional data is provided in Supplementary File 1.

### 3.4. The Coping Strategies in Relation to Sociodemographic Characteristics

In order to assess the influence of age on employed coping strategies, we performed the rho Spearman correlation test. In our population, older age slightly favored religious coping and correlated with a decrease in all of the other coping strategies, with the exception of behavioral disengagement, positive reframing, and denial, for which no statistically significant correlations were found. 

The U Mann–Whitney range test revealed that female participants were significantly more likely to cope with the use of self-blame, emotional and informational support, positive reframing, venting, denial, and behavioral disengagement. Considering the classification of coping strategies, they were also more likely than male participants to employ emotion-focused and avoidant coping.

The participants employed at the time of the study were significantly more often using active coping, planning, emotional and instrumental support, venting, self-distraction, substance use, and avoidant coping and had a tendency to problem-focused coping (*p* = 0.058) compared to the unemployed.

Based on analysis with the use of the Kruskall–Wallis test, we observed that participants with a middle or higher level of education were more likely to cope with the use of acceptance, self-blame, and emotion-focused coping. Additionally, a higher level of education also favored coping by planning, humor, positive reframing, and venting. 

Considering the amount of performed tests, the results of these analyses are provided in Supplementary File 1.

### 3.5. The Coping Strategies in Relation to Previous Psychiatric Treatment and Psychotherapy Use

We performed the U Mann–Whitney test to evaluate how previous psychiatric treatment and psychotherapy correlate with employed coping strategies (Table 4). We have noticed that participants who declared previous psychiatric treatment were more likely to report self-blaming, behavioral disengagement, substance use, and avoidant coping and less likely to use religious coping. Participants who reported previous psychotherapy were also more likely to cope with the use of the above-mentioned coping strategies (self-blame, behavioral disengagement, substance use, and avoidant coping) but were additionally more inclined to humor, venting, self-distraction, and emotion-focused coping. They were also less likely to report religious coping. 

### 3.6. The Coping Strategies in Relation to Time Since Diagnosis and Stress Level

In order to assess the influence of perceived level of stress and disease length on coping strategies, we used the Spearman rho correlation matrix (Table 5). Longer time since initial diagnosis correlated inversely with self-blaming, while the use of other coping strategies was not affected by this variable. However, higher levels of stress strongly correlated with self-blaming and showed a moderate correlation with avoidant coping, behavioral disengagement, denial, and venting. Weak positive associations were detected for substance use, self-distraction, and emotion-focused coping, while weak negative associations were observed for planning, positive reframing, active coping, emotional support, acceptance, and problem-focused coping.

## 4. Discussion

In our study, we describe and compare coping strategies used by four groups of chronically-ill patients. It is known that diagnosis of chronic disease poses a significant burden on mental health, resulting in long-term psychological distress manifested, among others, by anxiety or depressive symptoms [30,31,32]. The individual coping strategies used by people subjected to stress reflect one’s ability to adapt to new challenges and, in turn, influence mental health. The final outcome of coping with stress depends on various individual and external factors. While some of them, such as optimism or self-esteem, cannot be rapidly and easily targeted, research highlights the impact of others with the example of social support also in the circumstances of the global pandemic [30,33,34,35]. While the pandemic-related restrictions largely reduced access to social interactions and, therefore, social support increasing the risk of worsened mental health, the impact on the population of chronically-ill patients was even more deleterious. 

We report significant correlations of used coping strategies with sociodemographic factors, such as age, sex, employment status, education, or previous history of psychiatric treatment and psychological interventions. Additionally, we examine the differences between several groups of patients in employed coping strategies and indicate factors related to more adaptive coping. In another analysis conducted in our study group, we observed that almost half of the studied population of chronically-ill patients reported symptoms indicative of a depressive episode or anxiety. According to the General Health Questionnaire 28 (GHQ28) and PSS-10 scores, the highest intensity of symptoms was observed in patients with MS, while the kidney transplant recipients were the least likely to report complaints [19]. Further research in the MS group revealed over 80% of participants experienced high or moderate stress levels, which was more strongly correlated with social restrictions and interactions rather than the current condition resulting from the chronic disease [36]. In this comparison of coping strategies in four groups of chronically-ill patients, we link the previous results with potential higher sensitivity in patients with MS. We hypothesize that these patients are more likely to anticipate, observe and report even less substantial changes to their condition, which may be related to the progressive character of the disease. In our comparison, patients with MS declared higher use of each group of coping strategies than the three remaining groups. While a younger mean of age or lower comorbidity may explain some results, such as higher substance use or less religious coping, the general results profile emphasizes a high level of perceived stress and indicates the need to include psychoeducation or psychological interventions in the long-term treatment of this population. 

Chodkiewicz et al. report that female sex, younger age, and pre-existent disorders were associated with worse mental health outcomes and also with higher use of passive and avoidant coping strategies in healthy participants [37]. 

We observed similar effects with female participants declaring higher use of self-blame, venting, denial, and behavioral disengagement, while younger participants tended to report higher substance use, self-blame, venting, or denial compared to older participants. However, we observed that a longer time since initial diagnosis was linked to significantly decreased use of self-blame, indicating the potential pro-resilient effect of chronic disease. While recent research highlights the increase in psychoactive substance use in the general population due to the COVID-19 pandemic [38,39], the participants of our study declared low use of this coping strategy, which may be associated with the chronic illness and related treatment. 

Our results lie in agreement with previous research on lung transplant recipients and candidates, in which problem-focused coping (task-focused strategy) was also predominant [40]. However, in that population, the least common strategy was emotion-focused, whereas, in our study, all groups of patients were least likely to use avoidant coping. 

In regard to patients with MS, we obtained different results than the Turkish study by Altunan et al., which may be related to the sociocultural background [41]. While in both studies, patients with MS declared common use of active coping and acceptance; we rarely observed turning to religion as a relevant coping mechanism in response to the pandemic. 

Noteworthy, while our study design only allowed for singular data collection, it is important to mention potential findings regarding increased psychological resilience in chronically-ill patients in response to the COVID-19 pandemic. In the study by Davis et al., participants were found to report less personal suffering and more resilience [42]. In another study by Young et al., older adults were shown to experience less stress and use more problem-focused strategies and less avoidant coping in response to the COVID-19 pandemic compared to younger adults [43]. In our population of chronically-ill patients, older age slightly correlated with turning to religion and with a decrease in other coping strategies, with the exception of behavioral disengagement, positive reframing, and denial. These differences may be related to different sociocultural backgrounds but also to the previous diagnosis of chronic illness as a significant contributor to mental health. According to the study by Bonenkamp et al., no effect of the pandemic on the well-being of dialysed patients was found when measured with the use of health-related quality of life (HRQoL) questionnaires [44]. It seems that previous diagnoses of chronic somatic illness and the necessity of undergoing regular medical procedures may eventually lead to increased psychological resilience in conditions of chronic stress. The inverse correlation between time since diagnosis and the use of self-blaming in our study group may reflect one of the potential underlying mechanisms of this phenomenon. 

Interestingly, this finding may also be observed in people suffering from mental disorders. Previous manifestation of mental health complaints, such as depressive, obsessive-compulsive, or anxiety symptoms, was not found to predict poorer mental well-being in response to the COVID-19 pandemic development in a Dutch study comprising three large cohorts [45]. On the other hand, participants who previously did not report such symptoms were more likely to complain of them in response to the pandemic. In a Polish study by Talarowska et al., previous psychiatric treatment alongside non-adaptive coping strategies, such as denial, substance use, self-blame, behavioral disengagement, and venting, correlated with poorer mental health [46]. In our study, we observed that previous psychiatric treatment and psychotherapy strongly correlated with several disadaptive coping strategies, such as self-blaming, behavioral disengagement, substance use, and avoidant coping. We hypothesize that while both psychiatric patients and people choosing psychotherapy are more inclined to disadaptive coping strategies, the latter may also benefit from long-lasting changes to their personalities. Hence, additional more adaptive coping strategies, such as the use of humor and emotion-focused coping, can be distinguished in their behavior, which was reflected in our study results. 

Among numerous alarming reports regarding the general population, the direct impact of the COVID-19 pandemic on the treatment of patients with chronic diseases has been described in several reports. In a study by Singh et al., the interviewed chronically-ill patients declared they had avoided going to hospitals during the pandemic [5]. In Rwanda, almost half (44%) of the participants suffering from chronic diseases reported a lack of access to emergency care or medication and skipping clinical appointments [9]. In a study by Umucu and Lee, patients with chronic conditions who used self-distraction, denial, substance use, behavioral disengagement, venting, planning, religion, and self-blame were found to report higher levels of stress [47]. This finding lies in agreement with our study, in which the participants with higher PSS-10 scores employed similar coping strategies with the exception of planning and religion.

Another study by Girma et al. associated the use of active coping, denial, behavioral disengagement, self-blame, and turning to religion with higher PSS-10 scores in chronically-ill patients [48]. Our results confirm the correlations for self-blaming, behavioral disengagement, and denial. However, we did not observe an association between higher levels of perceived stress and the use of active or religious coping. 

Educating and encouraging adaptive coping strategies seems especially relevant in a population of chronically-ill patients considering the psychological burden and potential treatment discontinuation resulting from the aggravated stress. Among such interventions, behavioral activation, mindfulness practice, or acceptance-based coping are indicated in order to improve resilience and reduce stress intensity [49]. Moreover, taking advantage of available ways of telecommunication could facilitate mental state evaluation in vulnerable individuals and, in turn, allow for quicker interventions [50]. This approach could contribute to better compliance and improved treatment results in conditions of generalized stress-inducing factors. 

### Limitations

While our research reports data on coping in a relatively large group of chronically-ill patients, our results are also limited by several factors:

The studied groups were not matched in terms of sociodemographic characteristics, such as sex or education. 

Due to the global character of the COVID-19 pandemic, we were unable to recruit a control group. At the time of our study, singular participants were already individually affected by COVID-19 or quarantines. Hence, we were not able to analyze the impact of infections and individual restrictions on the outcomes of the study. 

We based our study on two data collection methods, paper questionnaires and an online version, which disabled us from providing a response rate to the study. Considering the large contribution of online questionnaires, we were also unable to confirm the medical diagnosis of patients with psoriasis and multiple sclerosis or to link obtained data with medical data such as laboratory results.

## 5. Conclusions

Compared to previous research, the studied groups of chronically-ill patients possess certain distinct characteristics in regard to coping strategies employed in response to the COVID-19 pandemic. While we confirm some of the previous findings made in the general population, such as the most common use of the problem-focused coping strategies and least frequent use of avoidant coping, or higher coping use correlating with higher perceived stress levels, we also highlight the impact of previously diagnosed chronic illness and its potentially pro-resilient effects on the coping profile. The characteristics of coping profiles in studied groups are dependent on numerous factors, including disease severity, prognosis, and daily burden. Group comparison identifies patients with a chronic neurological disease, such as multiple sclerosis, at higher risk of a less beneficial coping profile than kidney transplant recipients. Additionally, previous psychiatric treatment and psychotherapy both correlate with several less adaptive coping strategies, such as self-blaming, behavioral disengagement, substance use, and avoidant coping, while psychotherapy is also correlated with several beneficial outcomes, such as humor, venting, self-distraction, and emotion-focused coping. Our findings allow us to distinguish the individual and disease-related characteristics associated with adaptive and disadaptive coping in response to significant stress. Alongside telemedicine, further focus on education and early interventions in at-risk individuals is needed, and wide-targeted mental health programs are indicated in order to improve the mental health of patients suffering from chronic diseases.

## Figures and Tables

**Table 1:** Detailed sociodemographic data.

	Total n = 398	Psoriasis (P) n = 95 (23.9%)	Multiple Sclerosis (MS) n = 128 (32.2%)	Kidney Transplant (T) n = 102 (25.6%)	Dialysis (D) n = 73 (18.3%)	*p* Value
**Sex, female**	238 (59.8%)	58 (61%)	97 (76%)	49 (48%)	49 (48%)	*p* < 0.001
**Average Age (SD)**	45.74 (17.04)	40.19 (16.87)	35.63 (9.68)	51.59 (13.42)	63.40 (15.49)	*p* < 0.001 P vs. KT *p* < 0.0001 P vs. D *p* < 0.0001 MS vs. KT *p* < 0.0001 MS vs. D *p* < 0.0001 KT vs. D *p* < 0.005
**Illness duration in years** **(SD)**	10.71 (10.27)	16.65 (13.76)	12.27 (8.92)	7.77 (6.80)	3.52 (3.62)	*p* < 0.001 P vs. KT *p* < 0.0001 P vs. D *p* < 0.0001 MS vs. KT *p* < 0.001 MS vs. P *p* < 0.0001 KT vs. D *p* < 0.001
**Marital status** S—Single R—Relationship M—Married Se—Separated D—Divorced W—Widow(er)	S—69 (17.6%) R—72 (18.4%) M—205 (52.3%) Se—2 (0.5%) D—26 (6.6%) W—18 (4.6%)	S—21 (22.1%) R—20 (21.1%) M—41 (43.2%) Se—0 (0%) D—8 (8.4%) W—4 (4.2%)	S—27 (21.1%) R—36 (28.1%) M—58 (45.3%) Se—1 (0.8%) D—5 (3.9%) W—1 (0.8%)	S—17 (16.7%) R— 10 (9.8%) M—60 (58.8%) Se—0 (0%) D—10 (9.8%) W—2 (2%)	S—4 (5.5%) R—6 (8.2%) M—46 (63%) Se—1 (1.4%) D—3 (4.1%) W—11 (15.1%)	*p* < 0.001 P vs. D *p* < 0.002 MS vs. KT *p* < 0.04 MS vs. D *p* < 0.0001
**Psychiatric treatment**	73 (18.3%)	19 (20%)	38 (29.7%)	13 (12.7%)	3 (4.1%)	*p* < 0.0001
**Psychotherapy**	111 (28%)	29 (31%)	68 (53.1%)	11 (10.8%)	3 (4.1%)	*p* < 0.001
**Infection or quarantine of a close one**	6 (2%)	1 (1%)	4 (3,1%)	1 (1%)	0 (0%)	Not significant (NS)
**COVID 19 status**	Contact—2 (2%) Quarantine—12 (5%) Infection—2 (1%)	Contact—2 (2,1%) Quarantine—5 (5,3%) Infection—0 (0%)	Contact—no data Quarantine—7 (5,4%) Infection—2 (1,6%)	no data	no data	NS NS NS
**Stress level (PSS-10)** Low Moderate High	86 (21.8%) 246 (62.4%) 62 (15.7%)	31 (32.6%) 46 (48.4%) 16 (16.8%)	11 (8.6%) 85 (66.4%) 32 (25%)	29 (28.4%) 69 (67.6%) 4 (3.9%)	15 (20.5%) 46 (63%) 9 (12.3%)	

**Table 2:** Perceived stress and coping strategies.

	*R*	*M*	*SD*	*Mdn*	*Sk*	*Kurt*	*D*
Perceived Stress Scale (PSS-10)							
Perceived level of stress	3–37	18.95	7.22	18.00	0.15	−0.43	0.07 **
Coping Orientation to Problems Experienced Inventory (Brief-COPE)							
Acceptance	0–3	1.74	0.84	2.00	−0.49	−0.39	0.20 **
Active coping	0–3	1.84	0.84	2.00	−0.52	−0.29	0.17 **
Self-blame	0–3	1.08	0.87	1.00	0.49	−0.60	0.17 **
Planning	0–3	1.76	0.84	2.00	−0.42	−0.43	0.20 **
Humor	0–3	0.90	0.68	1.00	0.54	0.09	0.16 **
Emotional support	0–3	1.60	0.92	2.00	−0.15	−0.86	0.18 **
Use of informational support	0–3	1.50	0.87	1.50	−0.09	−0.72	0.13 **
Positive reframing	0–3	1.58	0.84	1.50	−0.16	−0.66	0.17 **
Venting	0–3	1.20	0.73	1.50	0.04	−0.58	0.16 **
Self-distraction	0–3	1.52	0.81	1.50	−0.17	−0.66	0.14 **
Denial	0–3	0.83	0.77	1.00	0.68	−0.26	0.18 **
Behavioral disengagement	0–3	0.78	0.74	0.50	0.70	−0.32	0.19 **
Substance use	0–3	0.33	0.64	0.00	2.20	4.66	0.42 **
Religion	0–3	0.90	0.99	0.50	0.72	−0.78	0.25 **
Problem-Focused Coping	0–3	1.67	0.69	1.75	−0.54	0.04	0.10 **
Emotion-Focused Coping	0–3	1.24	0.50	1.25	−0.35	0.48	0.07 **
Avoidant Coping	0–3	0.86	0.49	0.88	0.49	0.54	0.08 **

** *p* < 0.01; M—arithmetic mean; SD—standard deviation; Mdn—median; Sk—skewness; Kurt—kurtosis; D—Kolmogorov–Smirnov test result.

**Table 3:** Comparison of coping strategies between study subgroups.

	Psoriasis (P) (n = 95)		Multiple sclerosis (MS) (n = 128)		Kidney Transplant (T) (n = 102)		Dialysis (D) (n = 73)					
	*Mdn*	*Mrang*	*Mdn*	*Mrang*	*Mdn*	*Mrang*	*Mdn*	*Mrang*	*H(3)*	*p*	*ε^2^*	*Post-hoc*
a	1.50	166.42	2.00	223.93	2.00	206.85	2.00	189.45	15.28	0.002	0.038	a.**P** < a.**MS**; a.**P** < a.**T**
b	2.00	187.49	2.00	212.08	2.00	213.21	1.50	173.90	7.93	0.047	0.020	b.**D** < b.**MS**; b.**D** < b.**T**
c	1.00	198.41	1.00	225.46	1.00	171.11	1.00	195.06	13.35	0.004	0.034	c.**T** < c.**MS**
d	1.50	178.17	2.00	203.00	2.00	215.47	2.00	198.80	5.62	0.132	0.014	
e	1.00	197.66	1.00	218.13	1.00	185.13	1.00	189.31	5.85	0.119	0.015	
f	1.50	177.38	1.50	190.33	2.00	215.01	2.00	222.68	9.56	0.023	0.024	f.**P** < f.**D**
g	1.50	176.12	1.50	200.97	1.50	211.73	1.50	210.27	5.92	0.115	0.015	
h	1.50	178.48	2.00	220.18	1.50	199.25	1.50	190.93	8.02	0.046	0.020	h.**P** < h.**MS**
i	1.00	187.95	1.50	228.41	1.00	173.56	1.50	200.07	14.84	0.002	0.037	i.**P** < i.**MS**; i.**T** < i.**MS**
j	1.50	181.82	1.50	220.44	1.50	183.95	1.50	207.51	9.00	0.029	0.023	j.**P** < j.**MS**; j.**T** < j.**MS**
k	0.50	176.66	1.00	211.59	0.50	185.80	1.00	227.17	11.47	0.009	0.029	k.**P** < k.**D**; k.**T** < k.**D**
l	0.50	170.66	0.50	213.01	0.50	183.95	1.00	235.08	17.61	0.001	0.044	l.**P** < l.**MS**; l.**P** < l.**D**; l.**T** < l.**D**
m	0.00	227.59	0.00	227.32	0.00	163.09	0.00	165.05	48.94	<0.001	0.123	m.**T** < m.**P**; m.**T** < m.**MS**; m.**D** < m.**P**; m.**D** < m.**MS**
n	0.00	167.91	0.00	182.77	1.00	226.44	1.00	232.32	23.48	<0.001	0.059	n.**P** < n.**T**; n.**P** < n.**D**; n.**MS** < n.**T** n.**MS** < n.**D**
o	1.63	176.59	1.81	209.51	1.88	214.11	1.75	191.34	6.78	0.079	0.017	
p	1.17	174.92	1.33	215.76	1.25	194.11	1.33	210.51	7.82	0.050	0.020	p.**P** < p.**MS**
q	0.75	182.68	0.88	230.63	0.75	164.80	0.88	215.29	22.20	<0.001	0.056	q.**P** < q.**MS**; q.**T** < q.**MS** q.**T** < q.**D**

a—acceptance; b—active coping; c—self-blame; d—planning; e—humor; f—emotional support; g—use of informational support; h—positive reframing; i—venting; j—self-distraction; k—denial; l—behavioral disengagement; m—substance use; n—religion; o—problem-focused coping; p—emotion-focused coping; q—avoidant coping. Mdn—median; Mrang—mean range; H—Kruskall-Wallis test result; *p*—*p*-value; ε^2^—effect size.

**Table 4:** Coping strategies in relation to previous psychiatric treatment and psychotherapy.

	Psychiatric Treatment							Psychotherapy						
	No (n = 325)		Yes (n = 73)					No (n = 286)		Yes (n = 111)				
	*Mdn*	*Mrang*	*Mdn*	*Mrang*	*U*	*p*	*rg*	*Mdn*	*Mrang*	*Mdn*	*Mrang*	*U*	*p*	*rg*
Acceptance	2.00	202.50	1.50	186.15	10,888.00	0.262	0.08	2.00	197.21	2.00	203.62	15,360.50	0.609	0.03
Active coping	2.00	201.67	2.00	189.85	11,158.00	0.419	0.06	2.00	200.10	2.00	196.17	15,558.50	0.755	0.02
Self-blame	1.00	186.04	1.50	259.40	7489.50	<0.001	0.37	1.00	180.04	1.50	247.85	10,450.50	<0.001	0.34
Planning	2.00	201.30	2.00	191.47	11,276.00	0.499	0.05	2.00	199.25	2.00	198.36	15,802.00	0.943	0.00
Humor	1.00	199.94	1.00	197.53	11,718.50	0.868	0.01	1.00	192.90	1.00	214.73	14,127.00	0.081	0.11
Emotional support	2.00	201.26	1.50	191.68	11,291.50	0.511	0.05	2.00	201.48	1.50	192.62	15,164.50	0.480	0.04
Use of informational support	1.50	199.46	1.50	199.68	11,849.50	0.988	0.00	1.50	196.53	1.50	205.36	15,167.00	0.484	0.04
Positive reframing	1.50	203.77	1.50	180.49	10,475.00	0.111	0.12	1.50	198.28	1.50	200.86	15,666.00	0.837	0.01
Venting	1.00	197.08	1.50	210.27	11,076.00	0.366	0.07	1.00	190.41	1.50	221.14	13,415.50	0.014	0.15
Self-distraction	1.50	197.75	1.50	207.31	11,292.50	0.514	0.05	1.50	191.88	1.50	217.35	13,836.50	0.044	0.13
Denial	1.00	197.07	1.00	210.34	11,071.50	0.359	0.07	0.50	194.49	1.00	210.63	14,582.50	0.195	0.08
Behavioral disengagement	0.50	194.62	1.00	221.22	10,277.00	0.066	0.13	0.50	190.83	1.00	220.06	13,535.00	0.019	0.15
Substance use	0.00	194.00	0.00	223.97	10,076.50	0.010	0.15	0.00	190.84	0.00	220.01	13,540.50	0.004	0.15
Religion	1.00	206.14	0.00	169.92	9703.50	0.011	0.18	1.00	206.03	0.00	180.87	13,861.00	0.040	0.13
Problem-Focussed Coping	1.75	203.56	1.63	181.40	10,541.50	0.136	0.11	1.75	200.33	1.75	195.56	15,491.50	0.709	0.02
Emotion-Focussed Coping	1.25	198.40	1.25	204.38	11,506.50	0.688	0.03	1.25	192.84	1.33	214.88	14,110.50	0.085	0.11
Avoidant Coping	0.75	192.80	0.88	229.33	9685.00	0.014	0.18	0.75	186.64	0.88	230.84	12,339.00	0.001	0.22

Mdn—median; Mrang—mean range; U—U Mann-Whitney test result; *p*—*p*-value; rg—Glass’s estimator of effect size.

**Table 5:** Rho Spearman range correlations for coping strategies and disease length and coping strategies and perceived stress level.

	Disease Length (Years)	Perceived Stress Scale
Acceptance	−0.023	−0.109 *
Active coping	−0.010	−0.143 **
Self-blame	−0.149 *	0.522 **
Planning	−0.078	−0.218 **
Humor	0.044	−0.054
Emotional support	0.043	−0.115 *
Use of informational support	0.010	−0.022
Positive reframing	0.005	−0.198 **
Venting	−0.083	0.311 **
Self-distraction	−0.010	0.175 **
Denial	−0.050	0.390 **
Behavioral disengagement	−0.109	0.405 **
Substance use	−0.114	0.251 **
Religion	0.089	0.050
Problem-Focused Coping	0.005	−0.193 **
Emotion-Focused Coping	0.026	0.168 **
Avoidant Coping	−0.119	0.473 **

* *p* < 0.05; ** *p* < 0.01.

## Data Availability

The datasets used and/or analyzed during the current study are available from the corresponding author upon reasonable request.

## Supplementary Materials

The following supporting information can be downloaded at: https://www.mdpi.com/article/10.3390/ijerph20064814/s1, Supplementary File 1.
