# Supplementary material for: Coping of Chronically-Ill Patients during the COVID-19 Pandemic: Comparison between Four Groups
Source: Int J Environ Res Public Health. 2023 Mar 9;20(6):4814. doi: 10.3390/ijerph20064814 (PMC10049622; doi:10.3390/ijerph20064814)
Supplement: Supplementary file 1 [file ijerph-20-04814-s001.zip › ijerph-2249402-supplementary.pdf]

International Journal of
Environmental Research and Public Health

MDPI

Supplementary file 1

Rho Spearman range correlations for coping strategies in relation to age. .

	Age
Acceptance	−0.147 **
Active coping	−0.172 **
Self-blame	−0.291 **
Planning	−0.104 *
Humor	−0.172 **
Emotional support	−0.119 *
Use of informational support	−0.162 **
Positive reframing	−0.093
Venting	−0.276 **
Self-distraction	−0.159 **
Denial	−0.028
Behavioral disengagement	0.010
Substance use	−0.296 **
Religion	0.166 **
Problem-Focused Coping	−0.151 **
Emotion-Focused Coping	−0.221 **
Avoidant Coping	−0.163 **

* $p < 0.05$; ** $p < 0.01$.

Coping strategies in relation to gender.

	female (n = 238)		male (n = 160)				
	Mdn	*Mrang*	*Mdn*	*Mrang*	*U*	*p*	*rg*
Acceptance	2.00	205.22	2.00	190.99	17678.50	0.216	0.07
Active coping	2.00	205.36	2.00	190.78	17645.50	0.206	0.07
Self-blame	1.00	215.50	1.00	175.70	15232.50	0.001	0.20
Planning	2.00	201.31	2.00	196.81	18609.00	0.695	0.02
Humor	1.00	197.26	1.00	202.83	18507.00	0.627	0.03
Emotional support	2.00	210.49	1.50	183.16	16425.00	0.018	0.14
Use of informational support	1.50	214.60	1.50	177.04	15446.50	0.001	0.19
Positive reframing	2.00	211.08	1.50	182.28	16284.50	0.013	0.14
Venting	1.50	223.44	1.00	163.89	13343.00	<0.001	0.30
Self-distraction	1.50	207.95	1.50	186.93	17029.00	0.069	0.11
Denial	1.00	214.57	0.50	177.09	15454.50	0.001	0.19
Behavioral disengagement	0.50	210.32	0.50	183.41	16465.50	0.018	0.14
Substance use	0.00	204.62	0.00	191.88	17821.00	0.166	0.06
Religion	1.00	205.06	0.50	191.23	17716.00	0.218	0.07
Problem-Focused Coping	1.75	208.81	1.75	185.65	16824.00	0.048	0.12
Emotion-Focused Coping	1.33	218.01	1.17	171.96	14633.50	<0.001	0.23
Avoidant Coping	0.88	216.20	0.75	174.66	15065.00	< 0.001	0.21

Mdn – median; Mrang – mean range; U – U Mann-Whitney test result; *p* – *p*-value; rg – Glass's estimator of effect size.

Coping strategies in relation to employment status.

	employed (n = 31)		unemployed (n = 26)				
	Mdn	*Mrang*	*Mdn*	*Mrang*	*U*	*p*	*rg*
Acceptance	1.50	32.08	1.25	25.33	307.50	0.119	0.24
Active coping	2.00	32.73	1.50	24.56	287.50	0.057	0.29
Self-blame	1.50	32.08	1.00	25.33	307.50	0.121	0.24
Planning	2.00	34.81	1.00	22.08	223.00	0.003	0.45
Humor	1.00	30.19	0.75	27.58	366.00	0.540	0.09
Emotional support	2.00	33.69	1.00	23.40	257.50	0.017	0.36
Use of informational support	1.50	33.58	1.25	23.54	261.00	0.020	0.35
Positive reframing	1.50	31.50	1.50	26.02	325.50	0.205	0.19
Venting	1.50	34.02	1.00	23.02	247.50	0.011	0.39
Self-distraction	2.00	33.26	1.25	23.92	271.00	0.032	0.33
Denial	1.00	29.98	1.00	27.83	372.50	0.613	0.08
Behavioral disengagement	0.50	30.37	0.50	27.37	360.50	0.481	0.11
Substance use	1.00	33.47	0.00	23.67	264.50	0.017	0.34
Religion	0.00	27.56	0.00	30.71	358.50	0.421	0.11
Problem-Focused Coping	1.63	32.81	1.50	24.46	285.00	0.058	0.29
Emotion-Focused Coping	1.33	32.50	1.21	24.83	294.50	0.081	0.27
Avoidant Coping	1.13	33.71	0.88	23.38	257.00	0.019	0.36

Mdn – median; Mrang – mean range; U – U Mann-Whitney test result; *p* – *p*-value; rg – Glass's estimator of effect size.

Coping strategies in relation to education.

	I: primary (n = 25)		II: middle (n = 79)		III: higher (n = 119)					
	Mdn	*Mrang*	*Mdn*	*Mrang*	*Mdn*	*Mrang*	*H(2)*	*p*	ε^2	*Post-hoc*
a	0.50	62.16	2.00	110.46	2.00	123.50	19.53	< 0.001	0.088	a.I < a.II; a.I < a.III
b	1.50	90.64	2.00	109.47	2.00	118.17	4.08	0.130	0.018	
c	0.50	74.86	1.00	116.67	1.00	116.70	9.63	0.008	0.043	c.I < c.II; c.I < c.III
d	1.50	82.44	2.00	110.51	2.00	119.20	7.03	0.030	0.032	d.I < d.III
e	0.50	87.66	1.00	105.97	1.00	121.11	6.92	0.031	0.031	e.I < e.III
f	1.00	94.98	1.00	106.92	2.00	118.95	3.74	0.154	0.017	
g	1.00	85.06	1.50	113.11	1.50	116.92	5.24	0.073	0.024	
h	1.00	88.66	1.50	106.36	2.00	120.65	6.24	0.044	0.028	h.I < h.III
i	1.00	79.26	1.00	103.23	1.50	124.70	13.03	0.001	0.059	i.I < i.III; i.II < i.III
j	1.00	89.50	1.50	115.73	1.50	114.25	3.56	0.169	0.016	
k	1.00	106.68	1.00	111.36	1.00	113.54	0.26	0.877	0.001	
l	0.00	96.96	0.50	114.09	0.50	113.77	1.64	0.441	0.007	
m	0.00	108.20	0.00	105.70	0.00	116.98	1.97	0.374	0.009	
n	0.00	100.54	0.00	110.01	0.00	115.73	1.51	0.471	0.007	
o	1.38	89.24	1.75	108.96	1.75	118.80	4.62	0.099	0.021	
p	0.92	72.92	1.25	107.20	1.33	123.40	13.37	0.001	0.060	p.I < p.II; p.I < p.III
q	0.75	93.24	0.88	110.84	0.88	116.71	2.79	0.248	0.013	

a– acceptance; b– active coping; c– self-blame; d– planning; e– humor; f– emotional support; g– use of informational support; h– positive reframing; i– venting; j– self-distraction; k– denial; l– behavioral disengagement; m– substance use; n– religion; o– problem-focused coping; p– emotion-focused coping; q– avoidant coping. Mdn– median; Mrang– mean range; H– Kruskall-Wallis test result; *p*– *p*-value; ε^2– effect size.

Coping strategies in distinct subgroups – interactive approach.

		M	*SD*	*F*	*p*	*η*^{2}	*Post-hoc*
A	Problem-focused	1.67	0.69	377.65	0.000	0.482	B < A
B	Emotions-focused	1.24	0.50				C < A
C	Avoidant	0.86	0.49				C < B
I	Psoriasis	1.13	0.69	4.89	0.002	0.036	I < II
II	Multiple Scleroma	1.36	0.62				
III	Kidney Transplant	1.23	0.68				
IV	Dialyses	1.29	0.62				
I.A		1.51	0.78	3.94	0.002	0.015	I.B < I.A; I.C <
I.B		1.10	0.55				I.A; I.C < I.B;
I.C		0.79	0.51				II.B < II.A;
II.A		1.75	0.63				II.C < II.A;
II.B		1.33	0.45				II.C < II.B;
II.C		1.01	0.52				III.B < III.A;
III.A		1.75	0.67				III.C < III.A;
III.B		1.22	0.46				III.C < III.B;
III.C		0.71	0.41				IV.B < IV.A;
IV.A		1.64	0.64				IV.C < IV.A;
IV.B		1.29	0.52				IV.C < IV.B;
							I.A < II.A; I.A
							< III.A; I.B <
							II.B; I.B <
IV.C		0.92	0.48				IV.B; I.C <
							II.C; III.C <
							II.C; III.C <
							IV.C;

M – mean; SD – standard deviation; F– ANOVA test result; p – p-value; η^2 – eta-^{2} estimator of effect.
